# Mapping recombination cold spots in wheat via meiotic recombination in a large biparental population

**DOI:** 10.1093/g3journal/jkag097

**Published:** 2026-05-19

**Authors:** Maliheh Shaltouki-Rizi, Noah Dewitt, Mohsen Mohammadi

**Affiliations:** Department of Agronomy, Purdue University, 915 West State Street, West Lafayette, IN 47907, United States; School of Plant, Environmental and Soil Sciences, Louisiana State University, 104 Sturgis Hall, Baton Rouge, LA 70803, United States; Department of Agronomy, Purdue University, 915 West State Street, West Lafayette, IN 47907, United States

**Keywords:** genetic map resources, Marey map, hot and cold recombination regions, trapped genetic variations, linkage drag, cold spots, recombination, genetic mapping, wheat, RILs

## Abstract

Crossover (CO) recombination during meiosis is the fundamental driver of success in conventional plant breeding and a key determinant of genetic discoveries. Despite its importance, the fine-scale recombination landscape across the wheat genome remains partially characterized. Here, we constructed a dense genetic map by using 2,826 genotyping-by-sequencing SNP markers in a population of 345 F2:6 recombinant inbred lines, derived from a Penny x Yecora-Rojo cross. We constructed linkage maps spanning ∼3,507 cM across all 21 wheat chromosomes, and generated chromosome-wide recombination profiles and quantified local recombination rates in 5 Mb non-overlapping windows anchored to the reference genome. COs in wheat are overwhelmingly concentrated in distal and gene-rich regions, while the extensive pericentromeric cold spots span up to 77% of physical length. Low-recombination tracts (<0.1 cM/Mb) occupied 29.9 to 77.0% of chromosome physical length for all chromosomes. Average recombination rates ranged from 0.21 to 0.32 cM/Mb across the A subgenome, while local rates exceeded 5 cM/Mb in distal arms but dropped below 0.1 cM/Mb in centromeric regions. Chromosome 5A exhibited the longest genetic length (226 cM) but pronounced suppression in its subtelomeric and pericentromeric domains, whereas 4B showed the most extreme recombination desert, with 77% of its sequence nearly devoid of COs. These findings identified regions producing extended haplotype blocks and linkage drag in low-recombination regions that often constrain QTL resolution and limit access to allelic variation underlying key agronomic traits. The findings can also guide population design and inform strategies to redistribute COs through genetic, molecular, or epigenetic interventions by various genome rearrangement techniques in the future to unlock the hidden genetic diversity.

## Introduction

Crossover (CO) recombination is a significant genetic process that occurs during meiosis, where gametes are produced in sexually reproducing organisms. This process involves the exchange of genetic material between homologous chromosomes (Wang et al. 2021). This exchange creates new combinations of alleles by reshuffling of genetic material and is a major source of genetic variation (Lambing et al. 2017), and diversity, which is crucial for both evolution and selective breeding (Fayos et al. 2022). Leveraging recombination is an important tactic in assembling favorable allelic combinations across the genome (Lambing et al. 2017) or decoupling undesired linkages. The metrics used to characterize CO recombination are generally genetic and physical distances. Genetic distance between two loci is measured as recombination frequency between them and is expressed in cM, where one cM is equivalent to observing one recombinant in 100 progenies after one generation of meiosis. Physical distance, on the other hand, measures the actual base pair DNA nucleotide content between two loci, typically expressed in megabases (Mb) or kilobases (kb) (Tiwari et al. 2016). These two distances have complementary perspectives of genome architecture.

Recombination is not uniformly distributed along the genome. Instead, it occurs more frequently in certain regions known as recombination hotspots, while other regions known as cold spots or low-recombination regions (LRRs) have suppression of recombination (Schreiber et al. 2022). These LRRs are most often found within pericentromeric regions, which are distinct chromatin structure that flanks the centromere, often heterochromatin, and span over large physical ranges but correspond to short genetic distances. As a result, LRRs are thus hard to map or to decouple undesired genetic linkages in populations subject to selection (Fuentes et al. 2019). In plants, recombination hotspots may vary between a few hundred base pairs and several kilobases, and cold spots from a few kilobases to enormous regions of millions of base pairs (McConaughy et al. 2023). These cold spots are normally identified by comparing genetic and physical maps. Suppressed recombination is manifested as an elevated physical-to-genetic distance ratio expressed as kilobases per centiMorgan. For example, in barley, the centromere of chromosome 3H spans 58% of the physical length of the chromosome but accounts for only 5.5 cM of the total genetic map of the chromosome (Aliyeva-Schnorr et al. 2015). In rice, COs occur mainly in distal and gene-dense areas. whereas interstitial regions, located between the centromeric and telomeric domains, along with centromeric areas, show lower recombination (Marand et al. 2019).

From the breeding perspective, one of the most direct consequences of low recombination is the creation of long haplotype blocks where multiple loci are co-inherited in big blocks, minimizing the likelihood of decoupling unfavorable associations or pyramiding new favorable allele combinations (Fuentes et al. 2024). Such blocks are maintained unchanged due to the lack of COs, preventing allelic reshuffling and, therefore, are inaccessible to plant breeders. One very clear example is the Sub1 QTL for tolerance to submergence that was found in a cold spot with only 0.06 cM of distance between flanking markers. However, introgression into varieties like Swarna was only possible by screening of large populations facilitated by molecular markers (Septiningsih et al. 2009).

Though early genetic mapping efforts in the late 1990s and early 2000s, using the International Triticeae Mapping Initiative (ITMI) population, generated from the cross between Opata-85 and synthetic hexaploid W7984, provided valuable genetic resources for wheat (Röder et al. 1998; Somers et al. 2004), their foundation is limited by small population size and low-density SSR and RFLP markers, which are not amenable to fine-scale study of recombination. Despite decades of wheat genetics research, the cold recombination spots across the genome remain largely uncharacterized. Here, we characterize the relationship between physical and genetic maps by leveraging a relatively large recombinant inbred line population and the advent of the wheat genome assembly IWGSC RefSeq v1.0. In this manuscript, we explore the genome-wide pattern of recombination in all subgenomes and chromosomes of hexaploid wheat, determine LRRs, and discuss the implications of the recombination pattern for QTL mapping and breeding, considering issues of linkage drag, resolution boundaries, and introgression of traits.

## Material and methods

From a cross between Penny and Yecora Rojo, we developed a mapping population by single-seed descent method in a greenhouse and raised *n* = 345 F2-derived recombinant inbred lines (RILs) that were genotyped at F6 generation. Genotyping was performed using genotyping-by-sequencing (GBS) by using the *PstI-MspI* reduced representation libraries on leaf samples from single plants grown in the greenhouse. Sequencing was carried out on the Illumina NovaSeq platform at 176-plex multiplexing, using single-end 100-bp reads. Single-nucleotide polymorphism (SNP) calling and filtering were conducted using the TASSEL 5 GBSv2 pipeline, aligning reads to the IWGSC RefSeq v1.0 genome assembly. SNPs with >20% missing data were discarded, and missing data were imputed using the LDKNNi method (Money et al. 2015). Genetic maps were constructed by using ICIMapping software (Meng et al. 2015). Markers showing identical segregation patterns were grouped into co-segregation bins, and one representative marker per bin was retained for map construction. Distorted markers were removed, and recombination fractions were converted to genetic distance using the Kosambi mapping function (Kosambi 1943). Linkage groups were constructed by a threshold of LOD > 6. The final map consisted of 2,826 informative SNP markers distributed across all 21 wheat chromosomes. The longest genetic linkage groups identified per chromosome were maintained, and sporadic markers that were unlinked were omitted.

The accuracy of maps in pinpointing landmark agronomic loci was previously shown by Shaltouki-Rizi et al. (2024) on two phenotypes, i.e. flowering time and plant height, and in this report, we mapped the awn suppression locus and report its genetic and physical position in Supplementary Table 2 and Supplementary Fig. 1. The trait mapping provides an additional layer of confidence for the genotyping and mapping quality and the lack of segregation distortion. For QTL identification, inclusive composite interval mapping (ICIM) (Li et al. 2007) was used, with significance thresholds determined following Churchill and Doerge (1994), based on the maximum LOD scores obtained from 1,000 permutations using randomized phenotypic datasets, all implemented in ICIMmapping workflow.

To estimate recombination rates along chromosomes, mapped SNPs were anchored to their physical positions on the T. aestivum reference genome (IWGSC RefSeq v1.0). For each chromosome, cumulative genetic and physical distances were plotted to generate Marey maps (Chakravarti 1991). Marey maps plot cumulative genetic map distance cM against cumulative physical map distance (megabases) for each chromosome. Local recombination rates were estimated from LOWESS-smoothed Marey maps derived from marker-based genetic and physical positions, interpolated at 1-Mb resolution, and summarized in non-overlapping 5-Mb bins to capture broad chromosomal trends while reducing noise from individual marker intervals. Multi-megabase windows have been used in recombination landscape analyses of large cereal genomes, including a 5-Mb sliding-window approach in barley, and broader megabase-scale interval analyses in wheat, supporting the use of this scale for chromosome-level visualization rather than fine-scale hotspot detection (Dreissig et al. 2017; Gardiner et al. 2019). Regions with <0.1 cM/Mb were designated as cold spots (LRRs) consistent with previous wheat studies describing pericentromeric domains where recombination is strongly suppressed (Danguy des Déserts et al. 2021; Raz et al. 2021). Conversely, regions with 3 cM/Mb were classified as highly recombinogenic regions to highlight the upper tail of local CO activity in the present dataset. Because no universal absolute hotspot threshold is established across wheat recombination studies, this cutoff was used as a heuristic visualization criterion rather than a strict biological definition. These thresholds were chosen arbitrarily to highlight the extremes of recombination activity within our dataset. To evaluate the robustness of these classifications, we examined modest alternative thresholds for both low- and high-recombination regions and observed that the broad chromosome-scale recombination landscape remained qualitatively similar, although the exact number of extreme bins varied with cutoff stringency.

For chromosome-level summaries, physical length was defined as the physical coordinate of the last mapped marker on each chromosome, genetic length as the cumulative linkage-map distance, and mean recombination rate as genetic length divided by physical length. The proportion of the physical chromosome under suppressed recombination was calculated as the summed physical span of windows with a recombination rate <0.1 cM/Mb divided by chromosome length. Distal CO enrichment was quantified using a periphery-bias ratio (PBR), defined the proportion of total recombination occurring within the distal 20% of each chromosome length at both ends divided by the expected fraction under a uniform distribution, providing a measure of the preferential localization of COs toward chromosome ends where recombination is typically elevated due to pericentromeric suppression (Brazier and Glémin 2022).

To place the detected low-recombination intervals in chromosome context, the main plateau identified for each chromosome was overlapped with the published wheat chromosomal compartments R1, R2a, C, R2b, and R3. These compartments follow the wheat chromosome partitioning framework of Danguy des Déserts et al. (2021), in which R1 and R3 correspond to distal/telomeric regions, R2a and R2b correspond to proximal-interstitial/pericentromeric regions, and C corresponds to the centromeric region. For each chromosome, we recorded the compartments overlapping by the main low-recombination interval and identified the compartment showing the greatest overlap as the main chromosomal region associated with that plateau. For chromosome 5D, no main plateau was detected under the primary threshold; therefore, a shorter candidate interval was identified using a slightly relaxed slope threshold and is indicated accordingly.

Gene density was calculated from IWGSC RefSeq v1.0 annotations, summarized in the same 5-Mb windows, and paired with recombination windows by their physical midpoints. Relationships between recombination rate and gene density were evaluated using Spearman's rank correlation (ρ). All visualizations, including Marey maps, recombination rate profiles, and density plots, were generated in R (v4.3.2). The ggplot2, dplyr, scales, and zoo packages were used for graphical analysis and smoothing functions.

## Results

### Single-nucleotide polymorphism provides saturated and dense genome-wide marker coverage

The genetic maps were constructed by using 2,826 informative SNP markers distributed across the 21 wheat chromosomes (Supplementary File 2), covering a total genetic length of ∼3,506 cM (Supplementary File 1). Marker density and distribution varied across subgenomes and chromosomes. For example, the average adjacent marker distance on chromosome 2A is 1 cM, with 90% of the markers showing adjacent distances of less than 2.56 cM. The A subgenome carried 1,175 SNPs spanning ∼1,229 cM, the B subgenome contained 1,207 SNPs spanning ∼1,128 cM, and the D subgenome was relatively underrepresented, with 444 SNPs covering ∼1,148 cM. Average marker densities were 0.96, 1.04, and 0.35 SNPs per cM for the A, B, and D subgenomes, respectively, underscoring the lower resolution achieved in D due to fewer polymorphic loci. Variation was also evident at the chromosome level (Table 1).

**Table 1.** Distribution of molecular markers, length of linkage in each chromosome, and the map density calculated as the number of markers per cM of the linkage map.

	Chromosomes	1	2	3	4	5	6	7
A subgenome	Number of markers	174	166	192	99	242	103	199
	cM (Kosambi)	145.34	165.94	200.03	160.66	226.29	126.68	204.79
	Map density	1.2	1.0	1.0	0.6	1.1	0.8	1.0
B subgenome	Number of markers	149	247	298	113	148	98	154
	cM (Kosambi)	171.15	201.43	198.23	122.14	169.03	123.85	142.45
	Map density	0.9	1.2	1.5	0.9	0.9	0.79	1.1
D subgenome	Number of markers	67	66	74	34	61	44	98
	cM (Kosambi)	133.34	144.18	195.72	70.16	215.98	164.28	224.75
	Map density	0.5	0.5	0.4	0.48	0.3	0.3	0.4

**Fig. 1.**
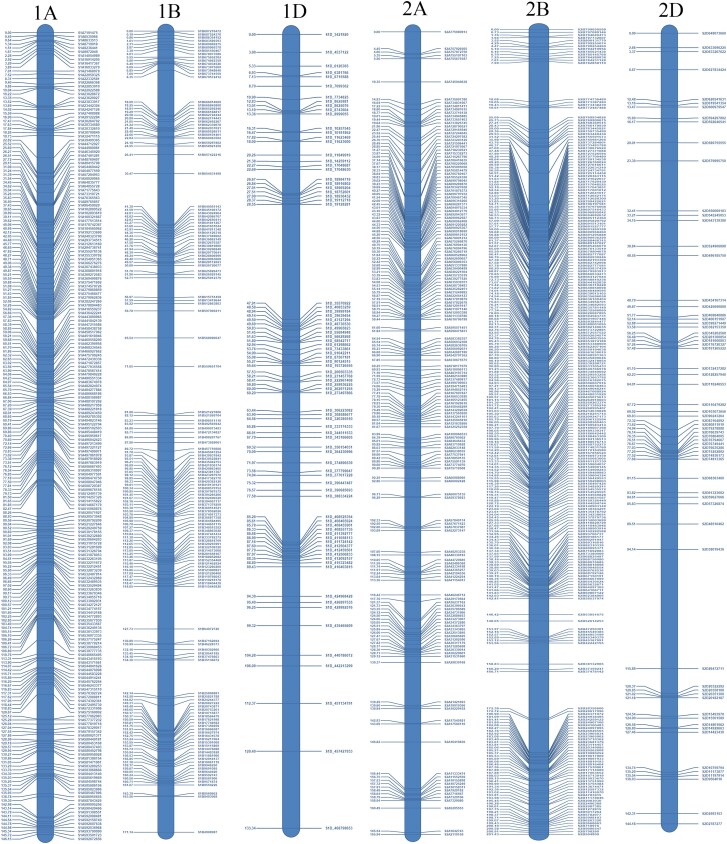
Genetic linkage map of wheat chromosome 1A, 1B, 1D, 2A, 2B, 2D constructed from the Penny × Yecora-Rojo RIL population. Each tick represents an SNP mapped according to recombination frequency. The scale at the right indicates genetic distance (cM) based on the Kosambi function. The maps of 21 wheat chromosomes are available in Supplementary File 3.

In the A subgenome, genetic lengths ranged from ∼127 cM (6A) to ∼226 cM (5A). The B subgenome ranged from ∼122 cM (6B) to ∼201 cM (2B), while the D subgenome ranged from ∼70 cM (4D) to ∼225 cM (7D). Chromosome 5A constituted the single longest linkage group, whereas 4D was the shortest. Marker density likewise varied, from a maximum of ∼1.5 SNPs/cM on 3B to a minimum of 0.27 SNPs/cM on 6D. Inter-marker intervals reflected this saturation, with adjacent markers spaced as little as 0.15 cM and rarely more than 5.7 cM apart (Table 1). An example of genetic maps drawn using ICIMapping software is shown in Fig. 1. (Genetic maps for all chromosomes are shown in Supplementary File 3).

### Recombination is strongly suppressed in pericentromeric regions and concentrated toward chromosome ends

Across the A-subgenome, chromosomes 1A–7A, we found that large physical chromosomes, ranging from ∼593 to 776 Mb, translated into relatively short genetic maps of only ∼126–226 cM (Table 2). This disparity reflects the highly uneven distribution of recombination along each chromosome. Average CO densities, knowing that this is after five generations of meiosis, were modest (0.21–0.32 cM/Mb), with broad regions of suppressed recombination surrounding the centromeres and elevated activity confined to the chromosome ends. In many cases, more than two-thirds of all COs were confined to just the terminal 5 to 10% of physical chromosome length, whereas expansive central regions spanning hundreds of megabases and harboring hundreds of genes with nearly no recombination. On chromosome 2A, a single pericentromeric cold spot spans hundreds of megabases. It contains approximately 300 genes, over which recombination is extremely rare, resulting in hundreds of genes trapped between successive CO events. In contrast, local recombination rates in distal, gene-rich intervals reached up to 5 to 7 cM/Mb, whereas rates dropped below 0.1 cM/Mb across pericentromeric regions. Although COs generally aligned with gene-dense regions, the correlation varied substantially among chromosomes. Spearman's ρ values ranged from 0.66 for 6A to 0.25 for 4A, reflecting that many genes are in regions where recombination is suppressed.

**Table 2.** Physical and genetic lengths and recombination metrics for wheat A-subgenome chromosomes.

Chromosome	Physical length (Mb)	Genetic length (cM)	Mean rate (cM/Mb)	Suppressed fraction (%)	Periphery-bias ratio	Spearman ρ (recomb vs gene density)
**1A**	593.8	145.34	0.24	34	4.45	0.62
**2A**	776	165.9	0.21	43	3.33	0.4
**3A**	749.28	200.0	0.27	37	3.57	0.72
**4A**	743.82	160.7	0.22	47	3.57	0.25
**5A**	709.86	226.3	0.32	40	3.72	0.42
**6A**	607.42	126.7	0.21	63	4.34	0.66
**7A**	734	204.8	0.28	51	3.58	0.47

Each chromosome’s physical size (assembly length) and genetic length (cM) are given, with the mean recombination rate (cM/Mb). Suppressed recombination is the fraction of physical length with very low crossover activity (centromere-proximal regions showing <0.1 cM/Mb). Periphery-bias ratio is the enrichment of recombination at the chromosome ends (ratio of the crossover fraction in the distal 20% of length vs that physical proportion). Spearman's ρ is the rank correlation between local recombination rate and gene density (negative intergenic distance).

**Fig. 2.**
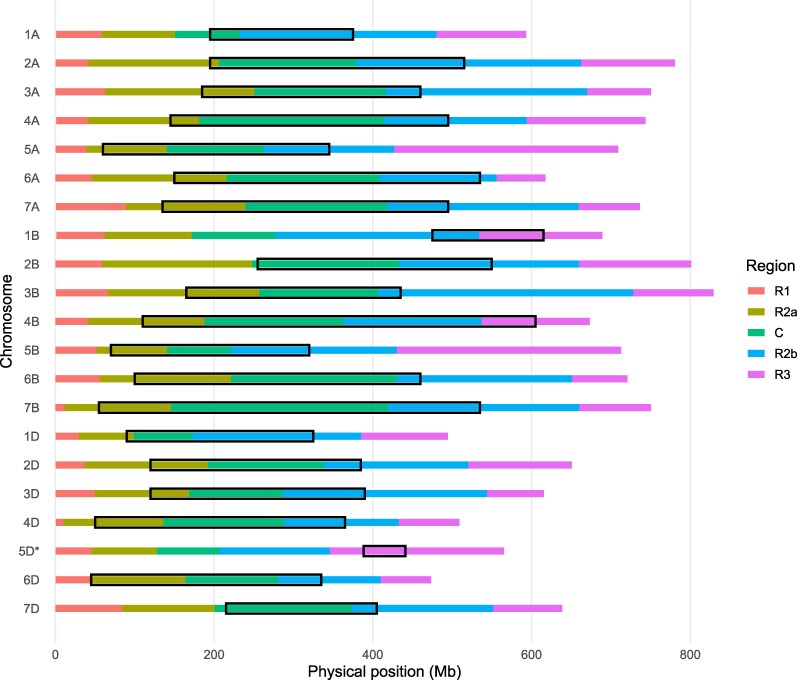
Physical positions of the main low-recombination intervals, shown by black outlined boxes, relative to wheat chromosomal compartments. Colored segments indicate the published wheat chromosomal compartments R1, R2a, C, R2b, and R3 as per Danguy des Déserts et al. (2021) definitions. Low recombination intervals are located in central chromosome domains, predominantly overlapping the C compartment and adjacent to R2 regions. The interval shown for chromosome 5D was included as a candidate plateau identified using a slightly relaxed threshold.

To clarify chromosome-specific CO distribution, we summarized the main low-recombination interval for each chromosome and mapped these intervals onto the published wheat chromosomal compartments R1, R2a, C, R2b, and R3 (Supplementary Table 1; Fig. 2). Most main low-recombination intervals were located in the central chromosome domains, predominantly overlapping the centromeric region (C) and adjacent R2 compartments, whereas distal regions (R1 and R3) were generally not the primary location of these intervals. Several chromosomes showed broad suppressed intervals spanning multiple adjacent compartments, most commonly R2a–C–R2b. Among the chromosomes with a detected main plateau, the largest overlap most often occurred in C, followed by R2a and R2b. Chromosome 5D differed from the others in that no main plateau was detected under the primary threshold; however, a shorter candidate interval was identified at 388.39 to 441.03 Mb under a slightly relaxed threshold.

### The saturated map enabled localization of awn suppression locus with high precision

The Yecora parent is awned, and Penny is awnless, resulting in segregation of awned and awnless phenotypes in the Penny × Yecora-Rojo progeny. A major QTL for awn suppression (LOD = ∼53) was detected on chromosome 5A in the distal long-arm region, with the peak located at approximately 699.9 Mb. The exact genetic and physical positions of the mapped interval are provided in Supplementary Table 2 and Supplementary Fig. 1. This interval is in ∼1.2 Mbp proximity of the awn inhibitor B1 (*Tipped1*), a major determinant of awn suppression on chromosome 5A, previously reported by the co-author DeWitt et al. (2020), providing a validation of the map quality in accurately deciphering genotype-to-phenotype relationship studies. In addition to this main QTL, we identified another adjacent locus located ∼8.3 Mbp apart (LOD = ∼16) (Supplementary Fig. 1, Supplementary Table 2).

### Genome-wide Marey maps reveal conserved chromosome-scale recombination patterns

**Fig. 3.**
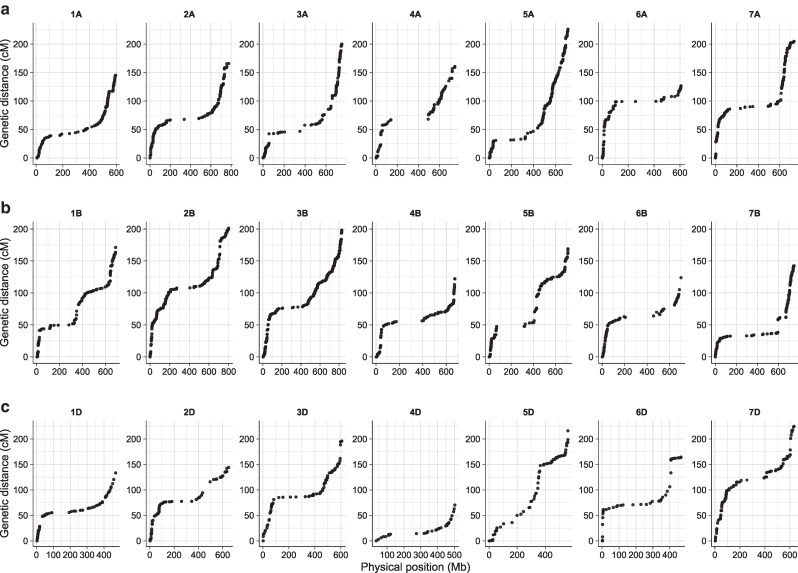
Marey maps show the relationship between physical and genetic distances across the 21 wheat chromosomes.(a) A-subgenome chromosomes, (b) B-subgenome chromosomes, and (c) D-subgenome chromosomes. Each panel plots genetic distance (cM) as a function of physical position (Mb) using marker positions only. The sigmoidal pattern reflects suppressed recombination in pericentromeric regions and elevated recombination toward the chromosomal arms. These patterns are consistent across homeologous chromosomes and highlight large recombination deserts in central genomic regions.

**Fig. 4.**
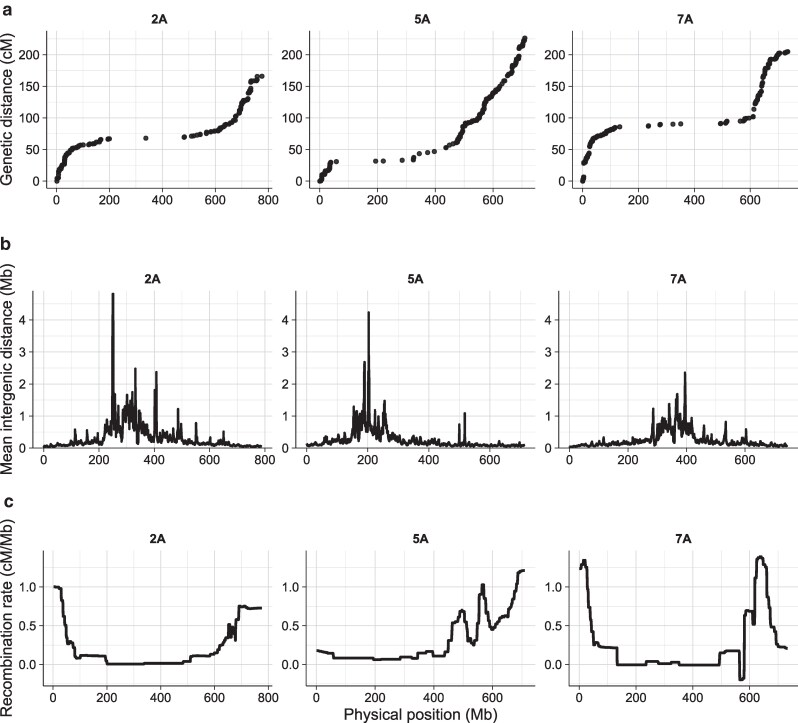
Recombination landscape and intergenic distance of wheat chromosomes 2A, 5A, and 7A. This figure summarizes three complementary views of recombination and genome structure across chromosomes 2A, 5A, and 7A. a) Marey maps show the relationship between physical position (Mb) and genetic position (cM). Each point represents a mapped marker, and the overall sigmoidal patterns reflect strong recombination polarization, with steep distal increases indicating crossover hotspots and extended pericentromeric plateaus marking recombination-suppressed regions. b) Local recombination rate profiles (1-Mb windows) further highlight the extreme distal enrichment of crossovers and broad mid-chromosomal cold regions where recombination approaches zero. Distal peaks mark localized recombination hotspots. c) intergenic distance, represented by mean intergenic distance (5-Mb centered windows), depicts the distribution of gene density along each chromosome. Large intergenic distances correspond to sparsely genic pericentromeric domains, while low intergenic distances at chromosome ends indicate gene-rich subtelomeric regions. The alignment between distal gene-dense regions and recombination hotspots, and the coincidence of sparse gene content with recombination deserts, reveals the tight coupling between genome structure and crossover localization in the wheat A-subgenome.

Genome-wide Marey maps illustrate this recombination architecture, with characteristic sigmoidal curves across all chromosomes (Fig. 3). The steep slopes at the telomeric regions reflect elevated CO activity, while the extended plateaus mark regions of suppressed recombination. The Marey maps show nearly identical topology in homeologous groups. These maps can be easily generated by readers via the genetic and physical map data provided in Supplementary File 1. A larger version of the three chromosomes is provided in Fig. 4.

A striking feature of hexaploid wheat is the differential recombination activity among its three subgenomes. The D subgenome exhibited consistently lower SNP density and shorter genetic map length relative to the A and B subgenomes, indicating reduced effective recombination and lower diversity across D-genome chromosomes.

### Chromosome-level relationships between recombination and intergenic distance

Chromosome-level patterns varied considerably. Chromosome 1A (∼593 Mb, 145 cM) showed the typical wheat pattern, a sigmoidal curve with distal recombination peaks and a strong positive correlation with gene density (ρ ≈ 0.62). Chromosome 2A (∼776 Mb, 166 cM) was highly polarized, with COs confined to the distal 10% of 2AL and a positive correlation with gene density (ρ ≈ 0.40), indicating that recombination still follows gene density in the distal regions despite large non-recombinant blocks in the interior. For chromosome 3A (∼750 Mb, 200 cM) about 36% of the chromosome length was suppressed, while distal peaks aligned moderately with gene-rich islands, producing a strong positive correlation (ρ ≈ 0.72). Chromosome 4A (∼743 Mb, 161 cM) showed one of the most extensive suppression patterns, with ∼47% of its sequence lacking recombination. COs were restricted to the tip of 4AL, while 4AS was entirely inactive, and a weak positive association with gene density was observed (ρ ≈ 0.25). Chromosome 5A (∼709 Mb, 226 cM) was distinctive for having the longest genetic length despite moderate physical size, but recombination was limited to subtelomeric regions and showed a positive correlation with gene density (ρ ≈ 0.42), reflecting alignment between gene-rich distal peaks and recombination activity. Chromosome 6A (∼607 Mb, 127 cM) was the smallest and least recombinogenic, with 63% of its length suppressed; yet its distal COs coincided with dense gene regions, producing a strong positive correlation (ρ ≈ 0.66). Finally, chromosome 7A (∼734 Mb, 205 cM) resembled 2A, with COs restricted to distal 7AL and a moderate positive correlation with gene density (ρ ≈ 0.47), highlighting the difficulty of breaking linkage blocks in its large recombination-suppressed region.

Figure 4 provides a detailed view of these patterns for chromosomes 2A, 5A, and 7A. In the top row, the Marey maps illustrate the characteristic sigmoidal pattern of wheat chromosomes, with long, flat segments across the pericentromeric regions and steep rises only at the chromosome ends. These flat segments correspond to large physical intervals where genetic distance does not increase, confirming the extent of recombination suppression. The distal steep slopes, most prominent on 2A and 7A, indicate that almost all COs are confined to the terminal regions of the long arms, consistent with the high periphery-bias ratios estimated for these chromosomes. The middle and bottom rows further clarify the structural basis for this recombination distribution. The intergenic-distance profiles (Fig. 4, middle panel) show large peaks in the central portions of each chromosome, marking extended gene-poor regions, indicating that the distance between two genes in the central region of chromosomes could reach as high as 4.83 Mb (in Chr 2A), while smaller intergenic distances toward the chromosome ends reflect gene-rich subtelomeric domains. These gene-rich regions align closely with the recombination hotspots observed in the local recombination rate plots. The bottom row shows that recombination rates, measured and expressed as cM per Mb, drop to near zero across the central >300 Mb of each chromosome, nearly one-third of the chromosome, while sharp peaks appear only at the distal ends. Together, these three panels in Fig. 4 demonstrate the tight coupling between chromatin structure, gene distribution, and recombination localization.

## Discussion

### Chromosome-scale organization of recombination in wheat

Our results are broadly consistent with previous studies showing that meiotic recombination in wheat is highly uneven along chromosomes, with CO activity concentrated toward distal chromosomal regions and strongly reduced in central domains. Earlier work has characterized these broad patterns using a range of mapping designs, including chromosome-specific analysis in segregating material together with diversity data, multi-parent populations, multiple recombinant inbred line populations, and LD-based inference from diversity panels. Examples include Darrier et al. (2017), Jordan et al. (2018), Gardiner et al. (2019), and Danguy des Déserts et al. (2021). This approach provides full genome coverage and a physically accurate view of gene and recombination spacing at both chromosome and subgenome levels. Our results confirm that bread wheat exhibits a highly polarized recombination landscape, with extensive cold spots across centromeric and pericentromeric regions and COs concentrated in distal, gene-rich telomeric regions, consistent with barley and maize (Wang et al. 2021). For example, on chromosome 3B, 90% of COs occur within just 14% of its physical length (Fayos et al. 2022), while pericentromeric regions comprising nearly two-thirds of the chromosome contribute less than 1% of recombination, leaving roughly 30% of wheat genes in recombination-poor regions (Erayman et al. 2004). Chromosome 4B is an extreme case, with nearly 77% of its physical length exhibiting recombination rates below 0.1 cM/Mb. Comparable patterns in maize and barley, where distal 20 to 30% of chromosomes account for most COs and pericentromeric heterochromatin remains largely inert (Kianian et al. 2018; Okagaki et al. 2018), suggest that large-genome grasses share strong distal enrichment of COs coupled with extensive recombination deserts near centromeres.

### High-resolution recombination mapping in a biparental population

Within this broader context, the contribution of the present study is not to redefine the general wheat recombination landscape, but to show how these established chromosome-scale patterns are expressed in the Penny × Yecora-Rojo biparental population. An advantage of this study is that it is based on a single, well-defined biparental mapping population, allowing recombination events to be interpreted directly within one meiotic framework and against one parental polymorphism background, without the additional complexity introduced by heterogeneous population structures. This enables chromosome-resolved estimation of major low-recombination intervals and their physical extent in this cross. At the same time, informative marker coverage in a biparental population is inherently constrained by the polymorphism segregating between the two parents, unlike multi-parent or diversity-panel resources that capture broader allelic diversity. We therefore use this population to define the physical extent of major low-recombination intervals, their overlap with published wheat chromosomal compartments, and the genomic regions in which variation may remain effectively trapped because of limited recombination and limited detectable sequence variation between the parents.

Earlier wheat linkage resources were extremely valuable, but most of them were much coarser than the present dataset for describing chromosome-scale recombination patterns. For example, the early microsatellite framework of Röder et al. (1998) mapped 279 loci, and the widely used consensus SSR map of Somers et al. (2004) included 1,235 loci spanning 2,569 cM. Other representative biparental studies from the SSR/AFLP era were also based on relatively modest marker densities and smaller populations, including 144 F_2_ with 227 markers over 2,849 cM (Thanh et al. 2013), 185 DH lines with 167 loci (Huang et al. 2006), and an intraspecific SSR map with 464 loci spanning 3,441 cM and ∼86% genome coverage (Torada et al. 2006). By comparison, our study used 2,826 mapped SNPs across 345 RILs, with average marker densities of 0.96, 1.04, and 0.35 SNPs/cM for the A, B, and D subgenomes, respectively, providing substantially greater genome-wide marker coverage than earlier SSR/RFLP-era resources within a single-cross framework. Although later multi-population consensus SNP maps contain many more markers overall, the present dataset provides a physically anchored recombination landscape in one defined biparental background, which is especially useful for interpreting CO distribution and identifying LRRs in this cross. The implications of this genome organization are significant. Individual pericentromeric cold spots can span hundreds of megabases and encompass substantial numbers of genes. For instance, a single pericentromeric region on chromosome 2A contains ∼300 genes over which recombination is exceedingly rare limiting allelic reshuffling and fine-scale mapping.

### Subgenome asymmetry and effective recombination in hexaploid wheat

A striking feature of hexaploid wheat is the persistent asymmetry in recombination activity among its three subgenomes. Our results confirm that the D subgenome exhibits consistently lower SNP density and shorter genetic map length relative to the A and B subgenomes. Importantly, this pattern does not reflect an absence of meiotic COs but rather reduced effective recombination resulting from severely limited standing genetic diversity. Population-scale analyses have shown that the D genome harbors lower nucleotide diversity and an excess of rare alleles compared to the A and B genomes, consistent with its origin from a limited number of Aegilops tauschii ancestors during hexaploid wheat formation (Jordan et al. 2018). In contrast, the A and B genomes have accumulated additional allelic variation through introgression from sympatric wild tetraploid relatives. Under low polymorphism, the CO events that occur between highly similar haplotypes remain genetically undetectable, leading to reduced genetic length and slower linkage disequilibrium decay despite ongoing meiosis. This phenomenon is consistent with the highly uneven distribution of nucleotide diversity observed along D-genome chromosomes (Akhunov et al. 2010) and explains why recombination suppression in the D subgenome remains a fundamental constraint on genetic resolution and breeding progress.

### Consequences for gene mapping and QTL resolution

Validation of our recombination maps using a major awn suppression QTL on chromosome 5A further confirms their resolution and biological relevance. The detected QTL co-localizes precisely with the known position of the dominant awn inhibitor B1 (Tipped1) in a narrow distal segment of 5AL (∼688 to 705 Mb), consistent with previous biparental and association mapping (DeWitt et al. 2020; Niu et al. 2020). Notably, the awn suppression locus mapped outside the major central low-recombination interval on chromosome 5A and instead fell within the distal long-arm region, where recombination is relatively elevated. Its successful localization to the known B1 region is therefore consistent with the greater mapping resolution expected in recombination-active chromosome segments, in contrast to the broader and less resolvable intervals expected for loci embedded within large central cold regions. For gene discovery, this means subtelomeric regions provide high mapping resolution, whereas genes in central regions remain locked in large, non-recombinant blocks.

### Implications for breeding and genetic gain

Recombination landscape has far-reaching implications for genetic diversity and breeding efficiency. Extended LRRs tend to maintain large haplotype blocks that are inherited with limited reshuffling across generations. Our results show that these central chromosomal tracts, often spanning hundreds of megabases, contribute relatively little to total genetic map length. In wheat, such regions may still contain substantial numbers of genes; for example, a 135 Mb pericentromeric interval on chromosome 1D contains 587 predicted genes in IWGSC v1.0 (Cavalet-Giorsa et al. 2024). In a biparental population, poor resolution in these intervals should not be attributed to suppressed recombination alone. Because this study used reduced-representation multiplex GBS genotyping, marker-rich and marker-poor regions were observed across all chromosomes. In broad central chromosomal regions, the low number of informative markers likely reflects not only recombination suppression but also limited detectable sequence variation between the two parents, which is an inherent limitation of biparental populations with restricted parental diversity. At the same time, across all 21 chromosomes, both marker density and recombination were consistently concentrated toward distal/telomeric regions, whereas broad central regions contained few markers and showed strongly reduced recombination. The recurrence of this pattern genome-wide suggests that it reflects a biologically meaningful feature of chromosome structure and polymorphism distribution in this population. As a result, alleles in these regions remain difficult to separate, reducing QTL mapping resolution and limiting the efficiency of classical breeding approaches for breaking undesirable linkage. On chromosome 5A, the dominant awn inhibitor B1 (Tipped1) maps to a distal segment of 5AL, between 698 and 705 Mb based on the Chinese Spring genome and has been consistently resolved with high precision (Huang et al. 2020). Similarly, the reduced-height locus Rht12 lies within the extreme terminal region of 5AL, where elevated recombination facilitates fine mapping and introgression (Sun et al. 2019).

Many other agronomically important loci, however, reside in recombination-suppressed regions. The stripe rust resistance gene Yr34 on distal 5AL, Vrn-A1 and Qfhs.ifa-5A on central 5AL, and the C locus on chromosome 2D all lie within regions of reduced recombination, complicating fine mapping and allele separation (Chen et al. 2021; Kajla et al. 2023). In such regions, beneficial alleles are frequently inherited together with undesirable linked genes, resulting in persistent linkage drag (Huang et al. 2023). The breeding challenge due to low recombination can be illustrated by estimating the population size required to recover at least one recombinant between two tightly linked loci with 95% confidence, using the equation *N* = ln(1 − *P*)/ln(1 − r) (Falconer and Mackay 1996; Frisch et al. 1999). Our genome-wide recombination estimates provide a quantitative indication of r for any region across the chromosomes. For instance, when r = 0.01 (1 cM), the required population size is approximately *N* ≈ 300, which is feasible for most breeding programs. However, when recombination frequency drops to r = 0.001 (0.1 cM), a population of roughly 3,000 individuals would be needed to recover a single recombinant with 95% confidence. At r = 0.0001 (0.01 cM), this number escalates to nearly 30,000, far exceeding the size of typical RIL or backcross populations. These estimates confirm that conventional selection cannot efficiently break linkage drag in recombination-suppressed regions (Hospital 2005; Collard and Mackill 2008), underscoring the necessity of molecular or biotechnological approaches to enhance or redistribute cO events.

Recombination deserts also have direct consequences for wheat breeding because they restrict the amount of accessible allelic variation within large pericentromeric regions. Low-recombination intervals tend to maintain long, intact haplotypes over many generations, meaning that both favorable and unfavorable alleles are inherited together and cannot be separated through crossing. This phenomenon has been observed in other cereals. Domesticated barley shows substantially reduced diversity and extended haplotype blocks in pericentromeric regions compared with wild relatives (Dreissig et al. 2019) and similar patterns are evident in wheat. A well-known case is the region on chromosome 4B surrounding the semi-dwarfing gene Rht-B1, where strong selection has preserved long haplotypes across diverse germplasm, like selection acting in a recombination-poor background (Hao et al. 2020).

These examples illustrate how breeding-driven selection within recombination deserts can lock large chromosomal segments, limiting opportunities to separate linked loci and reducing the pool of usable variation in these regions. Thus, we might infer that the D sub-genome has more to gain (in terms of captured diversity) from wild-relative introgression because it started with less diversity and perhaps has fewer beneficial alleles fixed historically (He et al. 2019). Since the D subgenome (from the diploid donor) is known historically to have undergone a stronger bottleneck in bread wheat origin than the A and B subgenomes, which were part of the tetraploid donor, the differences in gene content and structural variation could mean D has less utilized variation or is more subject to introgression and structural rearrangement. Overcoming this challenge will require strategies to redistribute recombination, whether through manipulation of Ph1, environmental shifts that modestly alter CO positioning, or targeted genome editing of recombination regulators. The patterns documented here mirror those observed in other large-genome cereals such as barley and rye, in contrast to smaller genomes like rice or Arabidopsis where recombination is more evenly spread. Quantifying these landscapes at chromosome resolution provides biological insight into wheat genome organization and highlights genomic regions where limited recombination may restrict access to linked genetic variation in breeding populations.

## Limitations and future directions

The limitation of this study is that the recombination landscape was derived from a single biparental cross, Penny × Yecora-Rojo. Broad features of wheat recombination, including distal CO enrichment and strong pericentromeric suppression, are generally conserved, but local CO patterns can vary among genetic backgrounds. For example, Gardiner et al. (2019) showed that major CO-rich regions are often shared across populations, whereas Danguy des Déserts et al. (2021) found that local recombination patterns differ among diverging bread wheat populations. Jordan et al. (2018) also showed that the recombination rate is genetically variable in wheat. Therefore, this dataset is best viewed as a high-resolution recombination reference for the Penny × Yecora-Rojo cross rather than a universal wheat recombination map. Similar analyses in additional populations will be useful to test how stable local cold-region boundaries and CO intensity are across wheat backgrounds.

## Conclusion

This dataset provides a chromosome-scale view of recombination landscapes in bread wheat, where we draw a clear relationship between GBS-based genetic map and physical maps. Our results help distinguish genomic regions that are more accessible to reshuffling from those located in recombination-poor intervals where genetic variation may remain difficult to separate. In this way, the study provides a clearer picture of the relationship between physical position and local recombination patterns in the Penny × Yecora-Rojo population, which is relevant for interpreting linkage relationships, mapping resolution, and the accessibility of variation across the wheat genome. Because recombination estimates depend on detectable SNP polymorphism and this map was derived from a single biparental cross, the present resource should be viewed as specific to the Penny × Yecora-Rojo background. Additional populations will be needed to assess how general these local recombination patterns are across wheat germplasm.

## Supplementary Material

jkag097_Supplementary_Data

## Data Availability

All raw data and computational methods supporting the findings of this study and the development of map resources are provided and fully accessible within the article and its Supplementary Materials. Supplementary File 1 contains the physical and genetic distances for all chromosomes analyzed in this study. Supplementary File 2 includes the complete genotype dataset across all lines and all markers used for all analyses. Supplementary File 3 contains a graphical representation of the genetic maps generated using this genotype data. Additional methodological details and Supplementary Information are described in the main text and figures. Supplemental material available at G3 online.
