# Supplementary material for: Mapping recombination cold spots in wheat via meiotic recombination in a large biparental population
Source: G3 (Bethesda). 2026 May 19;16(7):jkag097. doi: 10.1093/g3journal/jkag097 (PMC13334167; doi:10.1093/g3journal/jkag097)
Supplement: jkag097_Supplementary_Data [file jkag097_supplementary_data.zip › Supplemental_Figure_1_G3-2026-406748.pdf]

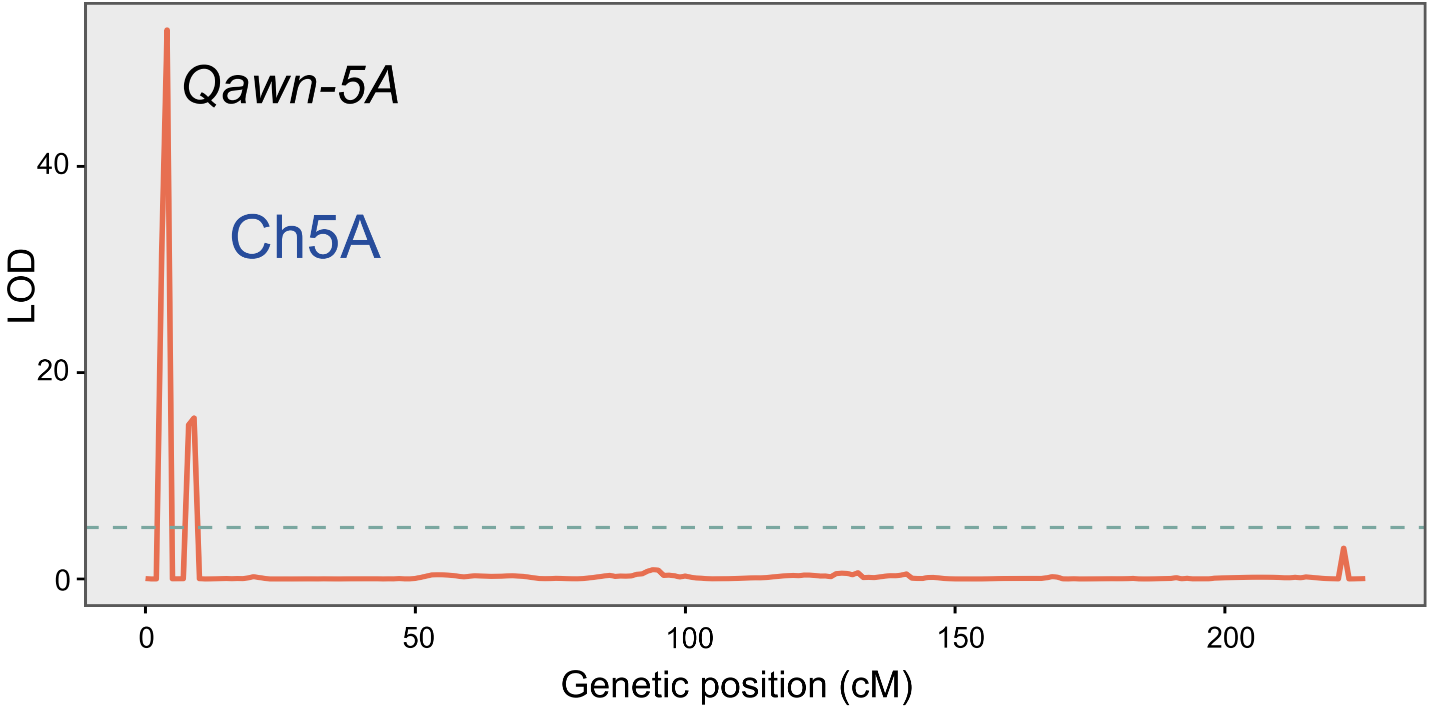

Figure S1. A major QTL for awn suppression detected on chromosome 5A in the Penny × Yecora Rojo population. Inclusive composite interval mapping identified a strong QTL on the distal long arm of chromosome 5A, with a peak at approximately 699.9 Mb and LOD > 50. The mapped interval is consistent with the reported position of the dominant awn inhibitor B1 (Tipped1) and supports the accuracy of the linkage map in distal recombination-rich regions.
