# Supplementary material for: Mapping recombination cold spots in wheat via meiotic recombination in a large biparental population
Source: G3 (Bethesda). 2026 May 19;16(7):jkag097. doi: 10.1093/g3journal/jkag097 (PMC13334167; doi:10.1093/g3journal/jkag097)
Supplement: jkag097_Supplementary_Data [file jkag097_supplementary_data.zip › Supplemental_Table_1_G3-2026-406748.docx]

**Table S1.** Chromosome-wise extent of low-recombination regions and their overlap with published wheat chromosomal compartments

| Chromosome | Chromosome length (Mb) | Total suppressed span (Mb) | Suppressed fraction (%) | Main LRR span (Mb) | Overlapping chromosomal compartments | Primary overlapping compartment |
| --- | --- | --- | --- | --- | --- | --- |
| 1A | 593 | 205 | 34.5 | 180 | R2b, C | R2b |
| 2A | 776 | 335 | 42.9 | 320 | C, R2b, R2a | C |
| 3A | 749 | 275 | 36.4 | 275 | C, R2a, R2b | C |
| 4A | 743 | 350 | 47.0 | 350 | C, R2b, R2a | C |
| 5A | 709 | 285 | 39.9 | 285 | C, R2b, R2a | C |
| 6A | 607 | 385 | 63.1 | 385 | C, R2b, R2a | C |
| 7A | 734 | 380 | 51.4 | 360 | C, R2a, R2b | C |
| 1B | 687 | 300 | 43.5 | 140 | R3, R2b | R3 |
| 2B | 800 | 295 | 36.6 | 295 | C, R2b, R2a | C |
| 3B | 829 | 270 | 32.3 | 270 | C, R2a, R2b | C |
| 4B | 673 | 520 | 77.0 | 495 | C, R2b, R2a, R3 | C |
| 5B | 712 | 355 | 49.7 | 250 | R2b, C, R2a | R2b |
| 6B | 701 | 365 | 51.8 | 360 | C, R2a, R2b | C |
| 7B | 745 | 480 | 64.4 | 480 | C, R2b, R2a | C |
| 1D | 468 | 235 | 50.0 | 235 | R2b, C, R2a | R2b |
| 2D | 649 | 270 | 41.2 | 265 | C, R2a, R2b | C |
| 3D | 607 | 290 | 47.5 | 270 | C, R2b, R2a | C |
| 4D | 502 | 320 | 63.4 | 315 | C, R2a, R2b | C |
| 5D* | 562 | NA | NA | 52.64 | R3 | R3 |
| 6D | 465 | 300 | 63.8 | 290 | C, R2a, R2b | C |
| 7D | 633 | 190 | 29.9 | 190 | C, R2b | C |

Chromosome length indicates the physical length (Mb) represented by mapped markers for each chromosome. Total suppressed span refers to the summed physical span of all windows with recombination rates <0.1 cM/Mb, and suppressed fraction (%) is the proportion of chromosome length occupied by these low-recombination windows. Main LRR span refers to the principal continuous low-recombination region identified on each chromosome for compartment-overlap analysis. Overlapping chromosomal compartments list all wheat chromosomal compartments intersected by the main LRR, whereas primary overlapping compartment indicates the compartment containing the largest proportion of that interval. Chromosomal compartments R1, R2a, C, R2b, and R3 follow the wheat chromosome partitioning framework of Danguy des Déserts et al. (2021), where R1 and R3 denote distal/telomeric regions, R2a and R2b denote proximal-interstitial/pericentromeric regions, and C denotes the centromeric region. For chromosome 5D, no main plateau was detected under the primary threshold; therefore, the reported interval was identified using a slightly relaxed slope threshold and is shown for descriptive comparison only. NA, not applicable.
