# Supplementary material for: Mapping recombination cold spots in wheat via meiotic recombination in a large biparental population
Source: G3 (Bethesda). 2026 May 19;16(7):jkag097. doi: 10.1093/g3journal/jkag097 (PMC13334167; doi:10.1093/g3journal/jkag097)
Supplement: jkag097_Supplementary_Data [file jkag097_supplementary_data.zip › Supplemental_Table_2_G3-2026-406748.docx]

**Table S2. Genetic and physical positions of awn-associated QTL peaks detected on chromosome 5A in the Penny × Yecora Rojo population**

| **Trait** | **chromosome** | **Peak position (cM)** | **Left marker** | **Right marker** | **Confidence interval (cM)** | **Physical interval (Mb)** | **LOD** |
| --- | --- | --- | --- | --- | --- | --- | --- |
| awn | 5A | 4.0 | 699797574 | 699851005 | 3.5–4.5 | 699.80–699.85 | 53.23 |
| awn | 5A | 9.0 | 693326887 | 691531434 | 7.5–9.5 | 691.53–693.33 | 15.62 |

Peak positions are reported in centimorgans (cM) from the ICIMapping scan. Left and right markers indicate the flanking markers associated with each detected peak, and physical intervals are shown in megabases (Mb) based on marker positions on the IWGSC RefSeq v1.0 wheat reference genome. The awn-associated QTL is included here as an independent validation of linkage-map quality and positional accuracy rather than as a central result of the study.
