# Supplementary figures and images for: Mapping recombination cold spots in wheat via meiotic recombination in a large biparental population
Source: G3 (Bethesda). 2026 May 19;16(7):jkag097. doi: 10.1093/g3journal/jkag097 (PMC13334167; doi:10.1093/g3journal/jkag097)

3A

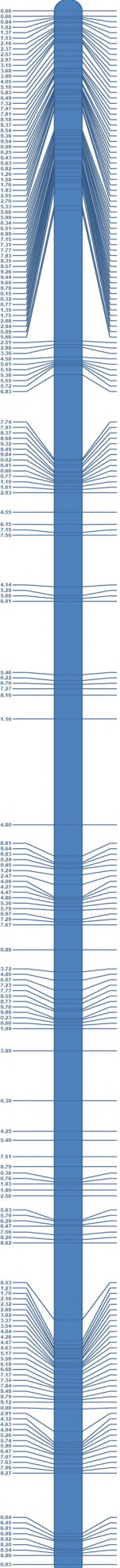

3B

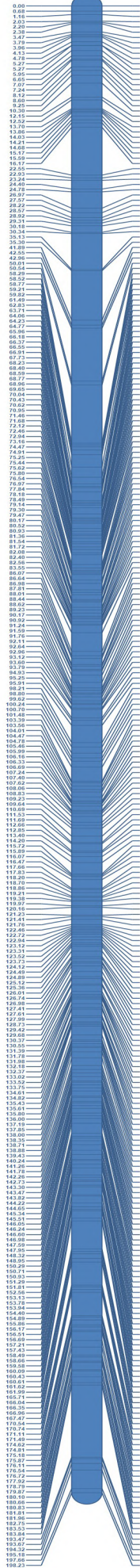

3D

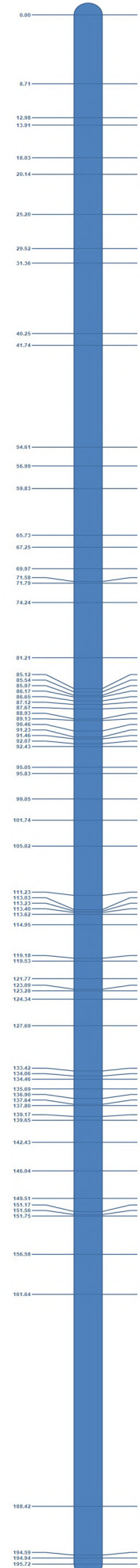

4A

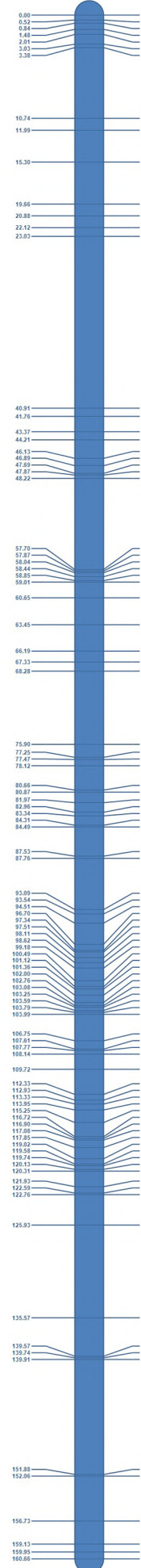

4B

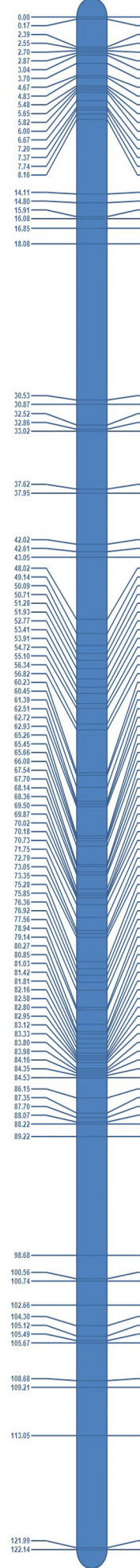

4D

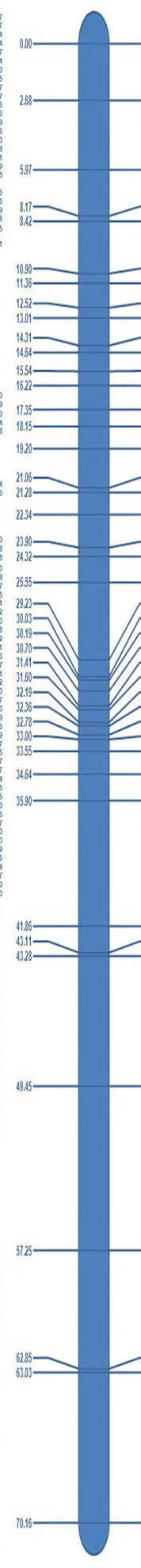

5A

5B

5D

6A

6B

6D

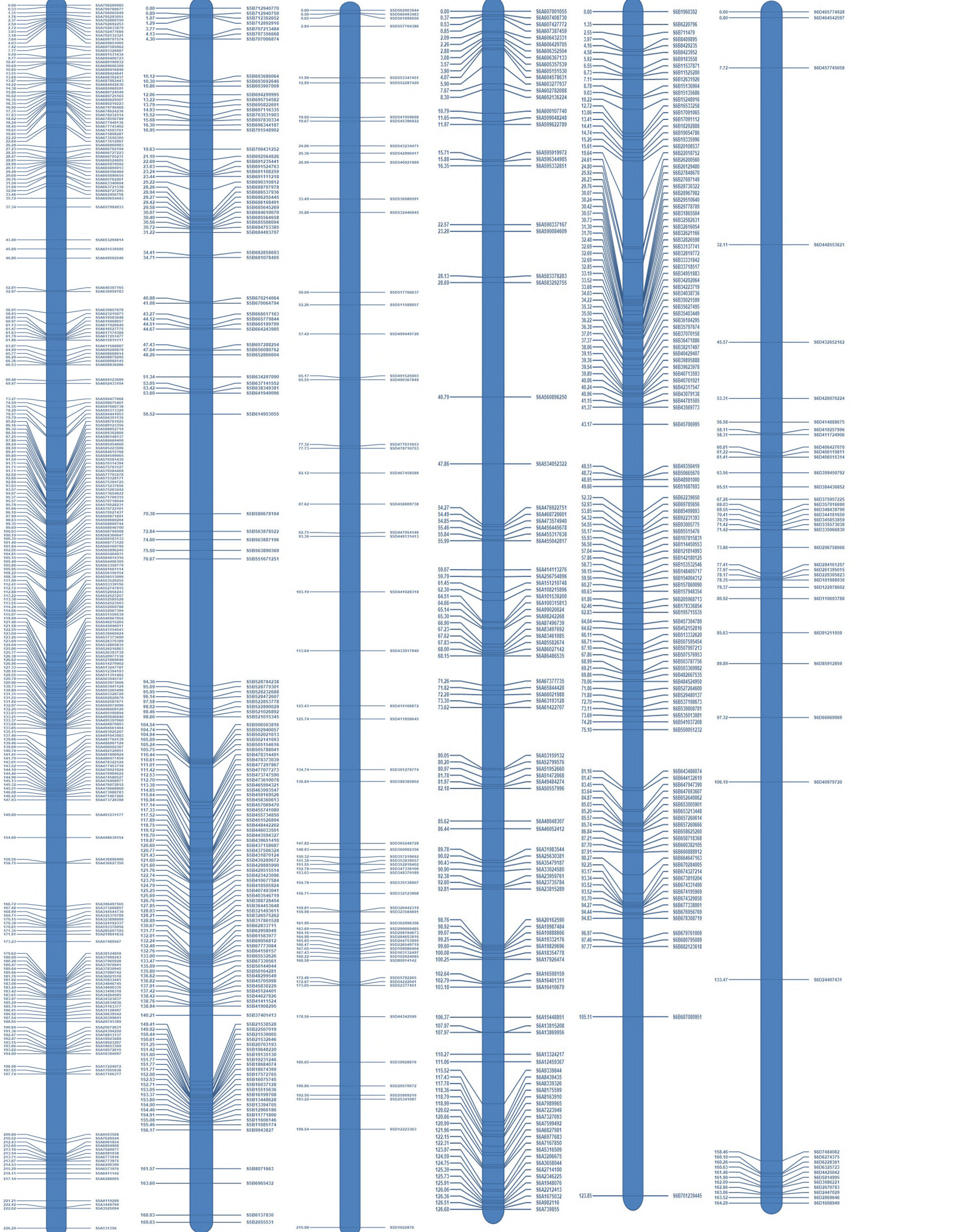

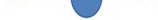

Supplement: jkag097_Supplementary_Data [file jkag097_supplementary_data.zip › Supplemental_File_3_G3-2026-406748.pdf]
